# Changing HCW attitudes: a case study of normalizing HIV service delivery in emergency departments

**DOI:** 10.1186/s12913-022-07942-2

**Published:** 2022-05-11

**Authors:** Aditi Rao, Victoria H. Chen, Sarah Hill, Steven J. Reynolds, Andrew D. Redd, David Stead, Christopher Hoffmann, Thomas C. Quinn, Bhakti Hansoti

**Affiliations:** 1 Department of International Health, The Johns Hopkins Bloomberg School of Public Health, Baltimore, MD USA; 2 Krieger School of Arts and Sciences, The Johns Hopkins University, Baltimore, MD USA; 3 Division of Intramural Research, National Institute of Allergy and Infectious Diseases, Bethesda, MD USA; 4 Department of Medicine, Johns Hopkins University School of Medicine, Baltimore, MD USA; 5 Department of Medicine, Faculty of Health Sciences, Walter Sisulu University, Mthatha, South Africa; 6 Department of Internal Medicine, Frere and Cecilia Makiwane Hospitals, Eastern Cape, East London, South Africa; 7 Department of Emergency Medicine, Johns Hopkins University School of Medicine, Baltimore, MD USA

**Keywords:** South Africa, HIV, HIV testing, Emergency Department, Men, HIV care cascade, Implementation science, Normalization process theory

## Abstract

**Background:**

Delays in the implementation of evidence-based practices are significant and ubiquitous, compromising health outcomes. Resistance to change is a key factor in hindering adoption and integration of new evidence-based interventions. This study seeks to understand the impact of exposure to HIV testing within a research context on provider attitudes towards HIV counselling and testing (HCT) in emergency departments (ED).

**Methods:**

This is a pre-and-post study design measuring the effect of a new ED-based HCT intervention, conducted by lay counsellors, on provider attitudes in Eastern Cape, South Africa. A validated, anonymized, 7-item survey was self-completed by routine care providers (physicians, nurses, and case managers). Questions were scored on a 5-point Likert scale with 5 consistently reflecting a positive attitude. Mean scores were calculated for each question and compared using a two-sample t-test to assess change in sample means for attitudes among providers surveyed before and after the intervention.

**Results:**

A total of 132 surveys were completed across three EDs. Majority of respondents were female (70.5%), 20–29 years old (37.9%), of African race (81.1%), nurses (39.4%), and practicing medicine for 0–4 years (37.9%). Pre-intervention, providers displayed a positive attitude towards ‘the benefit of offering ED-based HCT to patients’ (4.33), ‘the ED offering HCT’ (3.53), ‘all ED patients receiving HCT’ (3.42), ‘concern about patient reaction to HCT’ (3.26), and ‘comfort with disclosing HCT results’ (3.21); and a mildly negative attitude towards ‘only high-risk ED patients receiving HCT’ (2.68), and ‘the burden of offering HCT in a clinical environment’ (2.80). Post-intervention, provider attitudes improved significantly towards ‘all ED patients receiving HCT’ (3.86, *p* < 0.05), ‘only high-risk ED patients receiving HCT’ (2.30, *p* < 0.05), ‘the burden of offering HCT in a clinical environment’ (3.21, *p* < 0.05), and ‘comfort with disclosing HCT results’ (3.81, *p* < 0.05).

**Conclusions:**

Controlled exposure to new practices with a structured implementation period can shift attitudes beginning a process of practice normalization. In our study, we observed improvements in provider attitudes regarding the benefits of HCT and the burden of offering HCT to all patients in the ED. Research activities may have a role in mitigating resistance to change and supporting intervention adoption.

## Background

In the field of HIV, several well designed and funded interventions have failed to scale up and make the impact they planned due to the lack of buy-in from the health system and healthcare workforce [1]. While numerous evidence-based public health interventions and clinical treatment strategies exist, it can be challenging to implement and sustain them outside of controlled settings. Changing clinical practices and norms is challenging as it requires disturbing the status quo. Much clinical practice occurs in stable healthcare contexts and can be assumed to be habitual, wherein healthcare workers (HCW) have been described as “creatures of habit” and “resistant to change,” thus rendering existing clinical behaviors unlikely to be spontaneously reconsidered [2, 3]. Beliefs and perceptions held by HCWs have the potential to undermine the implementation and successful integration of interventions. A fear of the unknown and perceptions around how the status quo will be impacted by the changed behavior likely drives attitudes that hinder its acceptance. Additionally, in many cases the implementation of evidence-based interventions (EBIs) into practice aren’t supported with sufficient training or via supported process integration practices. Establishing a new normal also involves unlearning current practices and beliefs, and developing an understanding of the dynamic between new evidence-based practices and the existing context [4, 5].

South Africa faces the highest burden of HIV infection globally, with 7.5 million people (~ 20% of the global estimate) living with HIV and prevalence ranging from 12.6% to 27% across the country [6, 7]. As such, numerous measures have been taken by the national government to deliver EBIs focusing on HIV prevention and treatment, including routinely offered provider-initiated HIV counselling and testing (HCT) to all persons attending healthcare facilities as a standard component of medical care, including trauma, casualty, and specialty clinics, as per the South African National Strategic Plan on HIV, sexually transmitted infections and tuberculosis [8, 9]. However, much of the program’s focus has been directed to primary healthcare centers and antenatal facilities or to high-risk populations such as sex workers, men who have sex with men, injection drug users, and prisoners [10–12]. As a result, high-volume venues, such as EDs, are neglected, which remains the sole point of contact with the healthcare system for 28% of the country’s population [13].

The integration of HIV testing into health facilities has been pivotal to the goal of universal provision of testing. In the United States of America, routinization of HIV screening as a standard of care in EDs has been critical in shaping the national strategy for addressing HIV and has now been widely adopted across the country [14–16]. Previous studies in the Eastern Cape, South Africa, have also demonstrated a high burden of HIV in the ED with a prevalence of 25% and a new diagnosis rate of 7% [10, 17]. However, EDs are complex clinical environments, providers do not have formal HIV training, and routine HIV service delivery is not supported in this setting. Qualitative studies, including work by this team, has identified several potential barriers to HIV testing in the ED; concerns around confidentiality, increased burden of work for ED staff; high stigma among healthcare workers towards people with HIV; and lack of resources to provide social support or facilitate linkage to care [18]. We argue, the ED is a key clinical venue where patients missed by current HIV testing and treatment programs may be accessed, however, the listed barriers resulting in a lack of HCW buy-in impedes successful implementation and scale-up of ED-based testing.

Routinization of ED-based HCT testing will require overcoming cited barriers and a more reflective ‘normalization’ of practices [19]. There are several theoretical frameworks that seek to identify determinants of intervention adoption. This paper draws on the Normalization Process Theory (NPT), a theoretical model, which emphasizes that people change when their environment, or interactions with processes, change. NPT identifies, characterizes, and explains key factors that promote and inhibit the implementation and integration of complex healthcare interventions into everyday practice [20]. The theory has four core constructs; coherence (or sense-making); cognitive participation (or engagement); collective action (work done to enable the intervention to happen); and reflexive monitoring (formal and informal appraisal of the benefits and costs of the intervention) [21]. Drawing on these constructs NPT allows us to explore the processes through which complex interventions can be integrated and embedded as routine elements of clinical and organizational work in health care, making it applicable to assess if and why HCW attitudes shift after exposure to clinical research.

In this study we sought to understand the role of implementation research on provider attitudes to HCT and hypothesize that the process of bystander participation in research may normalize the practice and thus improve adoption of the intervention [21]. We proposed that controlled exposure to a new practice may also somewhat mitigate the fear of the unknown and perhaps decrease HCW stigma towards HCT service delivery. Here we use a validated survey instrument to quantify the attitudes of providers, pre-and-post exposure to the HIV research intervention, towards their acceptance and adoption of HIV service delivery within the ED and draw on NPT constructs to examine current thinking and approaches to inform large-scale behavior change.

## Methods

### Study overview

This research is nested within a three-year parent-project, the “Walter Sisulu Infectious Diseases Screening in the Emergency Department” (WISE) study, designed to implement a provider-initiated HCT intervention, and quantify the undiagnosed burden of HIV infection in the population presenting to EDs in the Eastern Cape, South Africa. Therein we sought to determine whether participation in- and exposure to- a cross-sectional HCT implementation study affects provider attitudes towards ED-based HCT. Previous findings from the WISE study have shown that the biggest challenges to the successful implementation of HIV testing have been providers’ perceived barriers to implementing a routine testing program [22]. By examining providers' survey responses at two time points (pre- and post-research intervention), this study seeks to identify and understand the shifts in provider attitudes affected by exposure to the WISE study, explained via key constructs of the NPT.

### Study setting

This study was conducted between September 2016 and September 2017 in three hospitals in the Eastern Cape province of South Africa: Frere Hospital (FH), Nelson Mandela Academic Hospital (NMAH), and Mthatha Regional Hospital (MRH). FH is a provincial, government-funded facility located in East London. NMAH and MRH are located in Mthatha and affiliated with Walter Sisulu University. NMAH is a large tertiary-care referral center with 24-h trauma services, and MRH is a district-level facility that provides services to walk-in patients as well as referrals from clinics. All three hospitals serve 100–150 patients per day from their surrounding 100 km catchment area.

HCT was not routinely provided in these EDs prior to this study. For the parent study, local research staff were hired and trained in rapid point-of-care HCT, Good Clinical Practice and data collection, and in sharing resources for subsequent linkage to care. During the research intervention, the research staff offered point-of-care HIV testing to all eligible patients presenting to the ED during the study period, 24- hours a day, in accordance with the South African National HIV Testing guidelines [9]. HCWs did not conduct HCT but were involved in identifying eligible patients, obtaining consent, and drawing blood samples for confirmatory tests. When applicable, HCWs were made aware of a patient’s HIV negative or positive status and aided study staff in disclosing the results to the patients and their families, as well as directing HIV positive patients to appropriate resources for linking to subsequent care. A more complete description of the intervention and methodology adopted is detailed elsewhere [22, 23]. Given the nature of the intervention, study staff engaged with HCWs during every patient interaction, and daily meetings with the charge nurse provided real-time feedback on how to strengthen HCT implementation but also raised concerns about the impact of study procedures on service delivery in the ED.

### Study population

A random sample of providers were enrolled in the study. All HCWs in the ED (physicians, registered nurses, nursing assistants) were made aware of the protocol for the HCT intervention and were informed of their respective roles and responsibilities. All HCWs were invited to participate in the study survey over a two-day time period, once at the beginning and once at the end of the six-week HCT intervention. Due to providers’ busy schedules and fluid work hours, they were encouraged to approach the study team when they were ready to participate.

### Survey instrument

Provider attitudes to ED-based HCT were assessed using a survey instrument, previously developed and validated by the study team [22]. The survey comprises of 14 questions, including seven demographic questions and seven questions assessing provider attitudes to HIV testing. To construct and validate this survey we previously completed exploratory factor analysis on a 53-question survey that focused on attitudes to HIV testing within health facilities, administered to 132 providers in the Eastern Cape region of South Africa. A full explanation of the survey development methodology is provided elsewhere [22]. The attitude questions were clustered into two factors, previously via factor analysis, ‘benefits of HIV testing’ and ‘comfort with providing HIV testing’.

### Data collection

Providers were approached to complete the survey at the start or end of their shift, or during breaks. Surveys were conducted in a private room to ensure as much confidentiality as possible. To encourage honest answers to potentially stigmatizing topics, all data were recorded anonymously with no identifying information. The surveys were conducted in English or Xhosa depending on provider preference and proficiency and were administered by the research team who recorded verbal results on an electronic handheld mobile tablet, using the Qualtrics © application (Qualtrics, Provo, UT). The survey included a brief introduction to the study and asked for verbal consent prior to recording any data. Questions were read out loud followed by answer options when applicable. Questions were repeated as needed, but not interpreted for the participant. Some providers preferred to read and input their answers into the tablet themselves.

Demographic data were recorded using pre-defined categories. Scoring for attitude questions was performed on a 5-point Likert scale with 1 being 'strongly disagree' and 5 being 'strongly agree’. Negatively worded questions were reversed in numeric value, so the number 5 consistently reflected a positive attitude.

### Data analysis

Analyses were performed using Microsoft Excel v.16.9 (Microsoft Inc.) and Stata v.14 (StataCorp, Tx). Tabulations were completed to determine mean attitudinal scores for individual survey items as well as for factor groupings, pre- and post- intervention, wherein a mean score of 3 represents an overall neutral attitude, while a mean score of more or less than 3 represents a positive or negative attitude, respectively. Additionally, a composite ‘attitude index’ was generated combining the seven individual questions to assess overall positive or negative attitudes towards ED-based HCT. The distribution of survey scores for each question, as well as the composite attitude index variable were checked for normality. The composite attitude index was then transformed into a binary variable, with responses ‘agree’ and ‘strongly agree’ used as indicators of a positive attitude, and ‘neutral,’ ‘disagree,’ and ‘strongly disagree’ used as indicators of a negative attitude.

A two-sample t-test was conducted to assess the change in sample means on the seven individual questions, factor groupings, and the composite attitude index on provider attitudes before and after the HCT intervention. Bivariate logistic regression was conducted to estimate the association between the composite attitude index and each provider characteristic, and multivariate logistic regression was completed to estimate the independent effect of provider characteristics, adjusting for others, on the composite attitude index, at baseline and after 6 weeks. A *p*-value of ≤ 0.05 was regarded as statistically significant.

### Ethical considerations

This study was approved by the Johns Hopkins University School of Medicine Institutional Review Board, the University of Cape Town Human Research Ethics Committee, and the Walter Sisulu University Human Research Ethics Committee. Verbal consent was obtained from all participants who enrolled in the study.

## Results

A total of 132 surveys (66 surveys each at baseline and after 6 weeks) were completed across the three sites [Table 1]. A majority of respondents were female (*n* = 93, 70.5%), between the ages of 20 and 29 years (*n* = 50, 37.9%), of African race (*n* = 107, 81.1%), and had been practicing medicine for 0 to 4 years (*n* = 50, 37.9%). Of the respondents, 52 (39.4%) were registered nurses, 45 (34.1%) were physicians, 19 (14.4%) were nursing assistants/practitioners, and 16 (12.1%) were case managers. Given the similarities in the overall sample population and the mean baseline attitude score by site, future analyses were pooled.

**Table 1:** Respondent characteristics by site, overall

Variable	Frere Hospital (*n* = 52) %	Nelson Mandela Academic Hospital (*n* = 50) %	Mthatha Regional Hospital (*n* = 30) %	Total (*n* = 132) %
**Age**				
20–29 years	15 (28.9)	17 (34.0)	18 (60.0)	50 (37.9)
30–39 years	10 (19.2)	15 (30.0)	6 (20.0)	31 (23.5)
≥ 40 years	27 (51.9)	17 (34.0)	6 (20.0)	50 (37.9)
**Sex**				
Male	11 (21.2)	19 (38.0)	8 (26.7)	38 (28.8)
Female	40 (76.9)	31 (62.0)	22 (73.3)	93 (70.5)
**Race**				
African	34 (65.4)	46 (92.0)	27 (90.0)	107 (81.1)
White	6 (11.5)	2 (4.0)	0 (0.0)	8 (6.1)
Coloured	12 (23.1)	2 (4.0)	3 (10.0)	17 (12.9)
**Highest level of education**				
< High School	3 (5.8)	0 (0.0)	0 (0.0)	3 (2.3)
Received high school diploma	11 (21.2)	1 (2.0)	1 (3.3)	13 (9.9)
≥ Bachelor’s degree	38 (73.1)	49 (98.0)	29 (96.7)	116 (87.9)
**Current position**				
Physician	14 (26.9)	17 (34.0)	14 (46.7)	45 (34.1)
Registered nurse	29 (55.8)	16 (32.0)	7 (23.3)	52 (39.4)
Nursing assistants / practitioners	5 (9.6)	10 (20.0)	4 (13.3)	19 (14.4)
Case managers	4 (7.7)	7 (14.0)	5 (16.7)	16 (12.1)
**Total years of practice**				
0–4 years	8 (15.4)	21 (42.0)	19 (63.3)	50 (37.9)
5–9 years	22 (42.3)	16 (32.0)	8 (26.7)	46 (34.9)
≥ 10 years	20 (38.5)	13 (26.0)	3 (10.0)	36 (27.3)
** Overall attitudes score**	3.16	3.60	3.62	3.44

^a^Data were missing for some variables therefore numbers do not always add to the total

### Attitudes to ED-based HCT

Before the HCT intervention, 63.6% (*n* = 42) of providers supported ED-based HCT, 90.9% (*n* = 60) felt patients will benefit from knowledge of their HIV status, 59.1% (*n* = 39) of providers expressed *all* ED patients should receive HCT, as opposed to 30.3% (*n* = 20) of providers who expressed only *high-risk* patients should receive HCT. Similarly, after the HCT intervention, 78.8% (*n* = 52) of providers supported ED-based HCT, 89.4% (*n* = 59) felt patients will benefit from knowledge of their HIV status, 77.3% (*n* = 51) of providers expressed *all* ED patients should receive HCT, as opposed to 18.2% (*n* = 12) of providers who expressed only *high-risk* patients should receive HCT.

Additionally, before the HCT intervention 42.4% (*n* = 28) of providers believed ED-based HCT will take up too much time and interfere with their job duties, and 57.6% (*n* = 38) of providers reported concern about patients’ negative reactions to HCT. And, after the HCT intervention 50% (*n* = 33) of providers believed ED-based HCT will take up too much time and interfere with their job duties, and 59.1% (*n* = 39) of providers reported concern about patients’ negative reactions to HCT.

### Independent correlates favoring ED-based HCT

Results of the bivariate and multivariate analysis of independent correlates favoring HCT in the ED, before and after the HCT intervention are summarized here. Before the HCT intervention, when adjusting for all other variables, physicians were more likely than nurses to demonstrate an overall positive attitude towards ED-based HCT (OR 0.42, 95% CI: 0.10–1.80, *p* < 0.05). Providers with 0–4 years of experience were more likely to demonstrate an overall positive attitude towards ED-based HCT than providers with 5–9 years of experience (OR 0.09, 95% CI: 0.01–1.11, *p* < 0.05). There was no significant correlation between providers’ age (30–39 years and ≥ 40 years vs. 20–29 years), sex (female vs. male), race (White and Coloured vs. African), and highest level of education (received high school diploma and bachelor’s degree or higher vs. high school or below). After the intervention, when adjusting for all other variables, there was no significant correlation between providers’ age (30–39 years and ≥ 40 years vs. 20–29 years), sex (female vs. male), race (White and Coloured vs. African), highest level of education (received high school diploma and bachelor’s degree or higher vs. high school or below), current position (registered nurse, nursing assistants/practitioners, and case managers vs. physicians), and total years of practice (5–9 years and ≥ 10 years vs. 0–4 years).

Table 2 summarizes mean scores for each question assessing provider attitudes before and after completion of the HCT research study in the ED. Overall, attitudes improved in all surveyed areas after the six-week research intervention. On a scale of 1 to 5, with 1 representing 'strongly disagree' and 5 representing 'strongly agree’, pre-intervention, providers displayed a strongly positive attitude towards ‘the benefit of offering ED-based HCT to patients’ (4.33); a mildly positive attitude towards the ‘the ED offering HCT’ (3.53), ‘all ED patients receiving HCT’ (3.42), ‘concern about patient reaction to HCT’ (3.26), and ‘comfort with disclosing HCT results’ (3.21); and a mildly negative attitude towards ‘only high-risk ED patients receiving HCT’ (2.68), and ‘the burden of offering HCT in a clinical environment’ (2.80). Post-intervention, provider attitudes improved significantly towards ‘all ED patients receiving HCT’ (3.86, *p* < 0.05), ‘only high-risk ED patients receiving HCT’ (2.30, *p* < 0.05), ‘the burden of offering HCT in a clinical environment’ (3.21, *p* < 0.05), and ‘comfort with disclosing HCT results’ (3.81, *p* < 0.05).

**Table 2:** Mean scores by question and factor

Variable	Pre-intervention stigma score (*n* = 66) Std. Dev.	Post-intervention stigma score (*n* = 66) Std. Dev.	*p-value*
**Question focus**			
ED should offer HCT	3.53 (1.23)	3.84 (1.12)	0.1371
ALL ED patients should receive HCT	3.42 (1.29)	3.86 (1.27)	0.0430*
Only high-risk ED patients should receive HCT	2.68 (1.08)	2.30 (0.99)	0.0363^*^
Benefit of offering ED-based HCT to patients	4.33 (0.73)	4.39 (0.68)	0.6447
Burden of offering HCT in a clinical setting	2.80 (1.15)	3.21 (1.33)	0.0440^*^
Concern about patient reaction to HCT	3.26 (1.33)	3.51 (1.06)	0.1663
Comfort with disclosing HCT results	3.21 (1.01)	3.81 (1.08)	0.0021^*^
**Factors**			
Perceived benefits of ED-based HCT	3.35 (0.61)	3.54 (0.55)	0.0770
Comfort in providing ED-based HCT	3.23 (0.86)	3.63 (0.96)	0.0159^*^
** Overall attitudes score**	3.32 (0.50)	3.56 (0.52)	0.0084^*^

^*^Significant at *p*-value < 0.05

When grouping the individual questions by their underlying constructs (derived through exploratory factor analysis, detailed elsewhere [22], we found pre-intervention, providers displayed a mildly positive attitude towards ‘perceived benefits of ED-based HCT’ (3.35), and ‘comfort in providing ED-based HCT’ (3.23), and post-intervention, provider attitudes improved significantly towards ‘comfort in providing ED-based HCT (3.63, *p* < 0.05). Furthermore, in comparing overall attitudes score, pre-intervention, providers displayed an overall positive attitude (3.32), which improved significantly post-intervention (3.56, *p* < 0.05).

### Normalization process theory framework

Drawing on NPT constructs, we examined the normalization of a diagnostics intervention, i.e., provider-initiated ED-based HCT, summarized below.

*Coherence* refers to the process of sensemaking, wherein a practice is made possible by a set of ideas about its meaning, uses, and utility. Here, individuals and organizations can establish coherence by defining the components of a practice and differentiating it from other, already established, practices. In our study, initially providers expressed reluctance in introducing HCT as part of the ED’s routine care pathway, suggesting it to be an additional burden on their time and resources, as compared to the existing practice of patients requesting HCT when desired or required. However, the perceived burden significantly improved post-intervention. The construct can also help understand why the proposed practice coheres (or fails to) with other clinical work. Of all providers surveyed, before and after the intervention, 90.2% of providers reported a benefit from knowing patients’ HIV status; as patients with HIV are more likely to develop secondary infections or have existing co-morbidities or underlying tuberculosis, resulting in a greater likelihood of experiencing adverse outcomes. Therefore, knowledge of patients’ HIV status allowed for better management of their clinical care.

*Cognitive Participation* examines how individuals and organizations engage with each other in enacting the newly adopted practice, wherein production and reproduction of a practice requires that actors collectively and repeatedly commit to it through the phases of initiation (agreeing on activities), enrolment (identifying roles and responsibilities), legitimation (believing in the validity of one’s involvement), and activation (committing to sustain the practice). In our study we found it is not enough for the leadership to recommend routine HCT or for nurses to be willing to conduct HCT at triage. In order for the practice to embed into routine care, all providers need to buy in to the practice, collectively contribute across stages of the clinical care pathway, and organize their roles accordingly. Before intervention, our study found; (a) physicians, and (b) providers practicing for 0–4 years were significantly more receptive to ED-based HCT compared to nurses and providers practicing for 5–9 years or more, respectively. These findings suggest unequal commitment and buy in to the practice among certain groups of providers, who can be appropriately targeted in subsequent interventions in order to build and sustain this new practice.

*Collective Action* describes the work individuals and organizations have to undertake to re-organize workflow and practices, wherein trust between administrators, physicians, nurses, and case managers need to be maintained in order to generate knowledge, build accountability, and maintain confidence in the team’s practices. Results from before the intervention suggest, 42.4% of providers believed introducing routine ED-based HCT will take too much time and adversely interfere with their job duties. Developing confidence in a new practice and subsequently ‘normalizing’ it relies substantially on effective division of labour, as well as the allocation of resources and execution of protocols and policies for managing the new practice. Therefore, in implementing the practice it can be hugely beneficial for teams to outline specific roles and responsibilities, and points in the care pathway where to enable the practice.

*Reflexive Monitoring* refers to the formal and informal appraisal of people’s actions towards the practice and the outcome, this may involve judgements about the utility and effectiveness of the practice and how it affects one individually and those around them, from which stems commitment to its conduct and performance. In our study, by part taking in routine ED-based HCT as part of research, providers were able to (i) assess the value of determining every patient’s HIV status and how it contributes to their overall care; (ii) understand existing norms and biases around HIV and how to effectively address it with patients, and (iii) feasibility of maintaining anonymity and confidentiality around the practice.

## Discussion

In our study, we saw slight improvements in both the providers’ assumed added burden of offering HCT, and their comfort and confidence with implementing ED-based HCT, following exposure to a research study that sought to implement HCT as part of the ED’s routine clinical care pathway.

Translation of national HIV testing guidelines to practice in the ED has been challenging worldwide [24–27]. Significant resistance to embedding HCT as routine practice in EDs has been raised by some in the emergency medicine community who are reluctant to re-negotiate existing practices in an already complex clinical setting [28, 29]. Despite the dissent among providers, there is a reassuringly high acceptance of ED-based HIV testing among patients [18, 30, 31]. Our previous work in this study population identified perceived operational barriers that an ED-based testing program would bring to the current work environment as a significant concern among providers [18]. Among the concerns raised, limited resources, discomfort with disclosing results, concerns with maintaining the confidentiality of results, patients’ reactions to a positive HIV test, ED financial struggles, and ED crowding, predominate [32, 33]. Many of these negative perceptions are anchored in providers’ existing knowledge of HIV testing provision across other health care facilities and their previous experiences in implementing interventions in the ED.

It is increasingly recognized that successful implementation of any complex intervention in a clinical setting begins with the willingness and commitment of providers to adopt such a program, followed by a strong intention to modify habitual practices [2]. And while provider support has been previously demonstrated as a key facilitator for the implementation of public health interventions in the ED (e.g., domestic violence and alcohol screening) [34–36], inherent in many social cognitive theories is also the assumption that providers have their beliefs, preferences, and intentions, but there are always additional social factors that promote or constrain particular expressions of agency [37]. Our study confirmed providers’ support and buy in as key stakeholders in the ED was crucial to the successful routinization of HCT, and additionally the NPT framework identified which actions facilitate or prevent the adoption of the newly proposed practices, and which key actors contributed and how, to its implementation, embedding, and integration. Furthermore, by exposing providers to the HCT program we were able to support their ability to make better sense of the true operational barriers to HCT implementation as well as enable engagement without making it mandatory or dictatory.

As difficult as it is for individuals and organizations to adopt new practices, equally important but considerably slower and more challenging may be the process of ‘de-innovation’, i.e., eliminating entrenched practices that previously made sense but are now less valuable, due to new evidence or competing approaches [38]. In our study, our aim was not only the implementation of a new practice, but also the de-implementation of deeply embedded and integrated patterns of interactions, expectations, and practices. The processes required to remove such established practices are not simply the reverse of those needed to initiate and adopt new ones. In more recent literature, the terminology of ‘de-innovation’ has become increasingly synonymous with unlearning [39]. There are numerous established strategies that can promote unlearning, such as education and persuasion, strategies to mitigate cognitive rigidity, systems engineering approaches to support alternative decision making, and use of social media [40, 41]. However, results of our study support the notion that controlled exposure to a new practice with an iterative design and continuous stakeholder buy-in can facilitate the process of unlearning and improve the adoption of a new intervention.

Longitudinal studies have already demonstrated the impact of policy change and normalization on longitudinal change in perceived barriers and benefits to testing in both patients and providers [42–44]. We have demonstrated that even short, discrete investments can have a significant role in not only changing the attitudes of providers, but also in improving the feasibility of adopting and scaling up new interventions.

In South Africa, a country with a complicated history around HIV/AIDS and significant investment in HIV service delivery, we suggest that research interventions may have a dual benefit. Not only is research necessary to understand the epidemiology of HIV and implementation of HIV service delivery, but also exposure to interventions within a research context may normalize HCW perceptions to HIV interventions. Research activities may thus have a significant role in supporting the acceptance, adoption, and scale-up of innovative HIV interventions that are required to curb the HIV epidemic.

## Limitations

This study has a limited sample size, capturing a small number of providers at the beginning and end of HIV testing intervention in the ED, and unfortunately, the participants were not paired due to the fluid nature of shift workers and their unpredictable schedules, thus it could mean that our results are representative of two different sample populations, opposed to a true change in attitudes of an individual provider. In addition, while the survey instrument utilized is validated, and normalization process theory was incorporated as part of the analytical framework, the survey itself could have discerned the impact of research activities across those constructs if the framework was part of the survey design, and provides an opportunity for future refinement of the survey tool.

## Conclusions

The results of this study support the theory that controlled exposure to a new practice, one that is learned and implemented iteratively, can facilitate the process of unlearning, where required, and improve the adoption of a new intervention. Understanding stakeholder attitudes and perceptions as well as structural factors of a given context through the lens of theory and practice can explain the failure of an apparently widely adopted and encouraged practice to become routinely incorporated, even in circumstances where professionals are favorably disposed to them and where significant material and political support was committed to them. Similar to other preventive programs, HCT cannot be easily layered onto current ED practices. The NPT model helps think about not just how its constructs can be introduced into planning and designing implementation processes, but also in asserting control over these practices which are influenced by other aspects of study design and context with uncontrollable outcomes. Implementation of small research projects not only benefit scientific inquiry but may go some way to changing perceptions, de-stigmatizing diseases and interventions, and prepare the system for change.

## Data Availability

The datasets used and/or analyzed during the current study are available from the corresponding author on reasonable request. The data are not publicly available due to their containing sensitive information that could compromise the privacy of research participants.

## Abbreviations

HIV: Human Immunodeficiency Virus
HCW: Healthcare Worker
ED: Emergency Department
HCT: HIV Counselling and Testing
NPT: Normalization Process Theory
WISE: Walter Sisulu Infectious Diseases Screening in the Emergency Department
FH: Frere Hospital
NMAH: Nelson Mandela Academic Hospital
MRH: Mthatha Regional Hospital
AIDS: Acquired Immune Deficiency Syndrome
